# Repercussions of Diagnostic Delay in Rare Diseases

**DOI:** 10.1002/jgc4.70258

**Published:** 2026-07-17

**Authors:** Luisa Rezende Batista, Luiza Fernandes Valente, Fernando Spina, Davi Said Gonçalves Celso, Israel Gomy, Christiane Mariotini‐Moura

**Affiliations:** ^1^ Federal University of Viçosa Viçosa Brazil; ^2^ Medical School of Ribeirão Preto, University of São Paulo Ribeirão Preto Brazil

**Keywords:** delayed diagnosis, neglected diseases, rare diseases

## Abstract

Rare diseases (RDs) are often subject to diagnostic delays due to their low prevalence, clinical variability, and limited professional awareness. This scoping review aimed to map the literature on these delays, examining their clinical, emotional, and socioeconomic consequences. Conducted in accordance with the PRISMA‐ScR guidelines, the review identified 23 studies published between 2010 and 2025. The included studies spanned 13 countries, with a notable concentration in Europe and increasing publication trends in recent years, reflecting growing international recognition of the challenges associated with delayed diagnosis. Across diverse study designs and disease contexts, commonly reported consequences included misdiagnosis and inappropriate treatment, psychological distress such as anxiety and frustration, disease progression, increased healthcare utilization, social isolation, reduced quality of life, and financial burden. These findings underscore the broad clinical and psychosocial impact experienced by patients during delayed diagnostic processes. Reducing diagnostic delay in RDs requires coordinated public health efforts, improved diagnostic infrastructure, and greater investment in professional training. Such efforts are essential to ensure earlier diagnosis, improve health outcomes and quality of life, as well as to enable timely access to genetic counseling to better support patients and families.

## Abstract (highlights)

**What is known about this topic:**

- The health repercussions of having a rare disease are vastly known throughout the system, individually linking them to each disease.
- The reasons for diagnostic delay are well known across rare diseases; however, most of the time, these factors are not managed correctly in clinical practice.

**What this paper adds to the topic:**

- This paper intends to understand the connection between the repercussions of diagnostic delay, such as social, economic and psychological.
- This paper also adds to the understanding of why such delays happen, digging into each selected study and exploring the findings associated with various rare diseases.

## 1 Introduction

The definition of a rare disease (RD) varies worldwide. In the United States (US), a RD is defined as one that affects fewer than 200,000 people, or 60 per 100,000 individuals (U.S. Food and Drug Administration 2020), while in the European Union (EU), it is considered rare when it affects fewer than 50 per 100,000 people (Eur‐Lex 2016). Brazil follows the World Health Organization (WHO) definition, which classifies a disease as rare when it affects fewer than 65 per 100,000 people. Despite their individual rarity, more than 8000 RDs collectively affect hundreds of millions globally, including at least 13 million people in Brazil alone (National Institutes of Health 2024; BVSMS 2014; Félix et al. 2022), thus representing a public health concern (Heuyer et al. 2017; Dharssi et al. 2017).

Approximately 80% of RDs are genetic and most are chronic (Global Genes 2024), often demanding prolonged and complex healthcare interactions. However, despite the existence of RD‐specific policies, the wide variety of these conditions, the challenges with insufficient information, limited access to specialized care, and the lengthy process of navigating the healthcare system often hinder timely diagnosis. Patients are often referred from one specialist to another, with long waiting times and unclear clinical pathways. These systemic and structural barriers contribute to what is widely known as “diagnostic odyssey” (Black et al. n.d.), described by Black et al. as “the medical journey traveled by patients with a rare disease (and their families) from initial disease recognition or onset of symptoms to a final diagnosis” (Black et al. 2015).

This prolonged journey is often characterized by multiple consultations, repeated diagnostic tests, and frequent misdiagnoses. Such delays are particularly harmful for RDs, as the majority are progressive and potentially life‐threatening (Isono et al. 2022). A study conducted in Australia among caregivers of children living with RDs reported not only clinical consequences but also psychological strain, including stress and anxiety (Anderson et al. 2013). These findings are echoed across literature, which consistently highlights the emotional toll experienced by both patients and families throughout this period of uncertainty (Benito‐Lozano et al. 2023).

Even after the diagnosis, patients must cope with the burden of the disease itself, which often significantly affects their functional capacity and social life. A EURORDIS survey revealed that more than half of patients or their families perceived the impact of the diseases as severe or very severe. Participants reported difficulties with daily activities due to the impairments of the diseases on motor and sensory functioning, as well as emotional distress, challenges in regulating behavior, and difficulties in managing finances (EURORDIS 2024a). These layered consequences illustrate how diagnostic delay not only postpones medical care but also exacerbates the social burden experienced by RDs patients.

In response to these challenges, the International Rare Diseases Research Consortium (IRDiRC) has set three goals to be accomplished in the decade between 2017 and 2027. The first goal focuses on reducing diagnostic delay, establishing that all patients with a suspected RD will be diagnosed within 1 year, with any diagnosis longer considered delayed. The second goal aims to improve treatment for RD patients by approving 1000 new therapies, the majority of which target diseases that currently have no approved treatment options. The final goal is to develop methodologies to assess the impact of diagnoses and therapies on RD patients (IRDiRC n.d.).

In alignment with these goals, countries have implemented strategies aimed at reducing these delays through improved awareness, infrastructure, and patient registries (Dharssi et al. 2017). In Brazil, the 2014 National Policy of Comprehensive Care for People with Rare Diseases marked a turning point, expanding access to multidisciplinary care, improving quality of life, and reducing morbidity and mortality (Félix et al. 2022; Ministério da Saúde n.d.). In addition, the National Neonatal Screening Program, in place since 2001, also facilitates early identification of six RDs, contributing to earlier intervention and better outcomes (Alves B/O/OM n.d.).

A recent scoping review by Lopes‐Júnior et al. (2022) acknowledges these advances and emphasizes Brazil's growing commitment to RD care. The study highlights a solid foundation of public policies, aligned with global priorities, including investment in research, specialized services, and social participation in policy‐making. Despite remaining challenges, such as reducing bureaucratic barriers and expanding access to services across all regions, the Brazilian model demonstrates meaningful strides in addressing the needs of patients with rare conditions.

Although substantial progress has been made both globally and nationally, a comprehensive synthesis of the actual repercussions of diagnostic delay remain limited. Given this complex landscape, this scoping review aims to map and synthesize the available evidence on the consequences of diagnostic delay in RDs, identifying key barriers and impacts on patient outcomes.

## 2 Methodology

This review was conducted in accordance with the Joanna Briggs Institute (JBI) Manual for Evidence Synthesis (Peters et al. 2020), which was influenced by the work of Arksey and O'Malley (2005) and PRISMA‐ScR guidelines. A protocol for this scoping review was previously published (Batista et al. 2024).

### 2.1 Identifying the Research Questions

To identify the research question for scoping reviews, the JBI recommends using the mnemonic “PCC,” which is also helpful for formulating the article title. PCC stands for Population, Concept, and Context.

Based on the PCC framework, the main research question of this review is: “What are the repercussions of diagnostic delay for RD patients in the global literature?”

Furthermore, other related questions also emerged as the study progressed, including: “What are the characteristics of the studies conducted to evaluate the repercussions of diagnostic delay in RDs?” and “What are the reasons reported for diagnostic delay of RDs by the authors or participants of the selected studies?” Data related to these secondary questions were also extracted from articles that answered the main research question.

### 2.2 Identifying Relevant Studies

Based on the research question, the search strategy included selecting specific keywords, phrases, and Boolean operators to ensure an exhaustive search. The final search was conducted on April 24th, 2025, and the results were systematically screened using well‐defined eligibility criteria, which included parameters such as the type of study, publication date, population, and relevance to the research question.

### 2.3 Setting a Search Strategy

To develop a comprehensive search strategy, an iterative process based on the PCC framework was conducted. Controlled vocabulary terms and free‐text keywords related to “rare diseases” and “diagnostic delay” were combined to maximize retrieval sensitivity while maintaining alignment with the research question. Search terms and synonyms were identified using the Medical Subject Headings (MeSH) vocabulary (NIH n.d.) and adapted when necessary according to the indexing systems of each database. Boolean operators (“AND” and “OR”) were applied to structure the conceptual blocks related to rare diseases and diagnostic delay.

Given the exploratory nature of scoping reviews, a broader and highly sensitive search strategy was intentionally adopted to minimize the risk of excluding potentially relevant studies. Specificity was subsequently achieved through predefined eligibility criteria, duplicate removal, multi‐stage screening, and independent reviewer assessment.

The final search strategy used was: (“Rare disease” OR “Rare diseases” OR “Orphan disease” OR “Orphan diseases”) AND (“Delayed diagnoses” OR “Delayed diagnosis” OR “Late diagnosis” OR “Late diagnoses” OR “Diagnosis delay”).

### 2.4 Choosing Databases

To ensure comprehensive coverage of the literature, five databases were selected based on their relevance to the study field of interest. These included both international and regional databases: PubMed, LILACS, Embase, Cochrane, and IBECS. A core search structure was developed and subsequently adapted for each database according to its indexing system, search syntax, and platform‐specific requirements.

Gray literature sources were also explored. Both governmental and non‐governmental websites that hold significant relevance to the study's area of interest were included in the search. This step aimed to capture unpublished data, reports, and policy documents that may not appear in traditional databases but could contribute with valuable insights. Furthermore, the authors reviewed the annals of the Brazilian Medical Genetic Congress (CBGM) from the past 5 years, which provided access to cutting‐edge research and presentations that had not yet been formally published (Table S1).

### 2.5 Eligibility Criteria

According to the JBI Manual for Evidence Synthesis (Peters et al. 2020), eligibility criteria should be based on the PCC mnemonic to properly address the research question.

Regarding the population (P), patients with any type of RD or description were included in this study. There were no exclusion criteria for the population.

The concept (C) used by this review was “repercussions of diagnostic delay.” Studies that refer to and explore the consequences of diagnostic delay in the lives of patients with rare diseases were included. Additionally, since the definition of diagnostic delay used by this review was based on the IRDiRC goal, only articles in which the diagnostic delay exceeded 1 year were included.

This review used global literature as its context (C), so any geographical location or study setting was included. No publication date limit was applied.

We considered primary empirical research studies and relevant review articles when they contributed synthesized evidence addressing the research question. Case series with three or fewer participants, single case reports, and dissertations were excluded. Articles were included only if they were full‐text, and written in English, Spanish, or Portuguese.

### 2.6 Data Charting and Synthesis

Researchers independently extracted key information from the selected studies and the CBGM abstract using standardized spreadsheets. These included publication details, disease focus, study type, and sample size. A second spreadsheet documented the consequences and causes of diagnostic delay, along with reported limitations. After independent data charting, the researchers reconciled their findings into a unified version for analysis.

## 3 Results

### 3.1 Study Selection

After database searches, 1472 articles were retrieved and imported into Rayyan (Ouzzani et al. 2016). After duplicate removal by two authors, 1368 unique titles remained.

After independent screening by two authors based on titles and abstracts, those not meeting the inclusion criteria or meeting any exclusion criteria were removed. Discrepancies were resolved by a third author, resulting in 94 articles selected for full‐text review.

Although the initial search retrieved a large number of records, most studies were excluded during title and abstract screening because they did not specifically address the repercussions of diagnostic delay in rare diseases or did not meet the predefined conceptual criteria. This staged screening process enabled the identification of studies directly aligned with the objectives of the review. The full‐text assessment identified 22 articles addressing the main research question.

Regarding the gray literature, none of the websites searched provided useful information. However, a review of 1709 abstracts of the CBGM annals resulted in 1 abstract that met the eligibility criteria and was included in this review. This screening process for this source was conducted by one author and subsequently revised by a second. Therefore, a total of 23 studies were included in the review.

**FIGURE 1:**
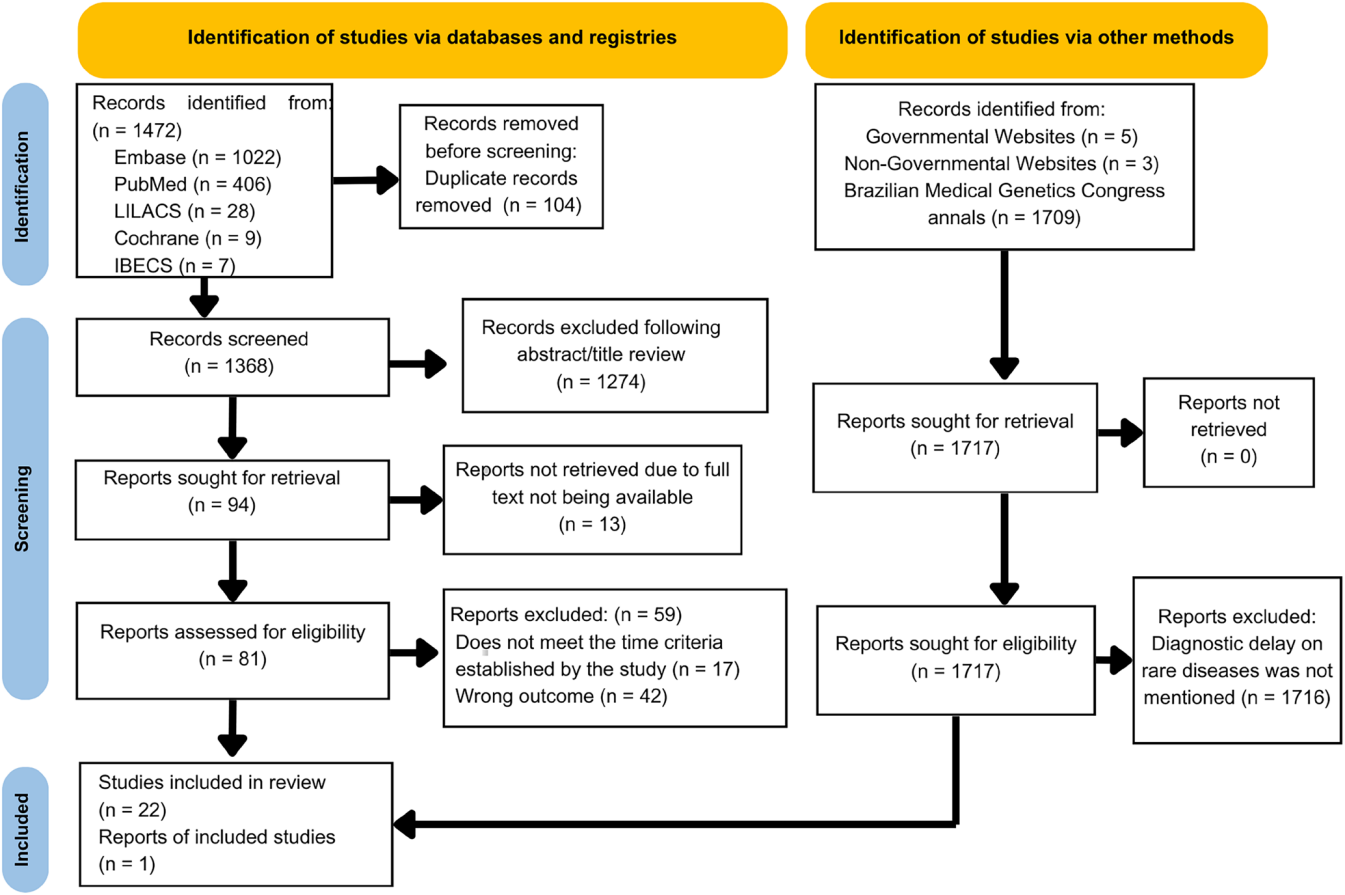
PRISMA flow diagram. All articles were selected by two independent reviewers.

This process was conducted according to the PRISMA‐ScR guidelines (Heuyer et al. 2017) and registered in a PRISMA flow diagram (Figure 1).

### 3.2 How and Where RDs Are Being Discussed

The 23 studies included in this review addressed RDs in two distinct ways. Nine studies analyzed the repercussions of diagnostic delays in RDs in general. The remaining 14 studies investigated the consequences of late diagnosis of specific RDs, namely: Hereditary Angioedema (HAE), Idiopathic Subglottic Stenosis (ISGS), Dopa‐Responsive Dystonia (DRD), Gaucher Disease (GD), Acromegaly, Mevalonate Kinase Deficiency (MKD), Pulmonary Alveolar Proteinosis (PAP), Hereditary Hemorrhagic Telangiectasia (HHT), Non‐Paraneoplastic Sensory Neuropathy (SN), Fabry Disease (FD), Sternocostoclavicular Hyperostosis (SCCH), Alpha‐Mannosidosis, Primary Immune Deficiency (PID), Sarcoidosis, Myositis and Myasthenia Gravis (Table 1).

**TABLE 1:** Presentation of information regarding the selected articles.

Authors/year	Country	Methodology/study design	Sample size (*N*)	Instrument	Disease studied
Benito‐Lozano et al. 2023	Spain	Cross‐sectional	524	Questionnaire	Rare diseases in general
Tanaka and Shimaoka 2023	Japan	Cross‐sectional	1000	Questionnaire	Rare diseases in general
Benito‐Lozano et al. 2022	Spain	Case–control	1216	Questionnaire	Rare diseases in general
Gimenez‐Lozano et al. 2022	Spain	Cross‐sectional	163	Questionnaire	Rare diseases in general
Isono et al. 2022	Japan	Qualitative	9	Semi‐structured interview	Hereditary Angioedema (HAE)
Berges et al. 2021	United States (US)	Historic cohort	124	Chart review (healthcare consultations)	Idiopathic Subglottic Stenosis (iSGS)
Páramo‐Rodríguez et al. 2023	Spain	Qualitative	25	Interview	Rare diseases in general
Rivera Gallego et al. 2021	Spain	Cross‐sectional	324	Healthcare consultation	Rare diseases in general
Kim et al. 2021	South Korea	Case‐series/Meta‐analysis	12;137	Healthcare consultation and database search	Dopa‐responsive dystonia (DRD)
Qi et al. 2021	China	Cross‐sectional	40 patients;49 caregivers	Questionnaire	Gaucher Disease (GD)
Wang et al. 2021	China	Cross‐sectional	447	Questionnaire/ 447 patients	Acromegaly
Gainotti et al. 2018	Italy	Review	N/A (Review)	Literature search/—	Rare diseases in general
Nunn 2017	United Kingdom (UK)	Letter to the editor	1	—	Rare diseases in general
Berody et al. 2015	France	Cross‐sectional	13	Questionnaire	Mevalonate kinase deficiency (MKD)
Ilkovich 2014	Russia	Historic cohort	68	Healthcare consultations	Pulmonary Alveolar Proteinosis (PAP)
Pierucci et al. 2012	Italy	Cross‐sectional	233	Questionnaire	Hereditary Hemorrhagic Telangiectasia (HHT)
Van der Kloot et al. 2010	The Netherlands	Cross‐sectional	52	Structured interview and questionnaire	Sternocostoclavicular hyperostosis (SCCH)
Rolim et al. 2019	Brazil	Cross‐sectional	48	Structured interview	Non‐paraneoplasic sensory neuropathies
Tanaca 2018	Brazil	Historic cohort	32	Chart review (healthcare consultations)	Fabry Disease
Faye et al. 2024	31 European Countries	Cross‐sectional	6507	Questionnaire	Rare diseases in general
Bai et al. 2025	China	Historic cohort	1692	Chart review	Acromegaly
Phillips et al. 2024	Australia	Qualitative	26	Semi‐structured interview	Myositis, Primary immune deficiency and Sarcoidosis
Corstés‐Vicente et al. 2024	5 European Countries: France, Germany, Italy, Spain and the UK	Cross‐sectional	387	Structured interview	Myasthenia Gravis

Given the diversity of the RDs included in the selected studies, Table S2 presents an overview of their reported prevalence according to major reference sources: Orphanet and National Organization for Rare Disorders (NORD) (Orphanet n.d.; National Organization for Rare Disorders 2015). It is important to note that prevalence figures may vary significantly depending on factors such as age group, ethnicity, geographical region, and the diagnostic criteria applied. In some cases, data were unavailable in one or more sources, indicated by “—”.

**FIGURE 2:**
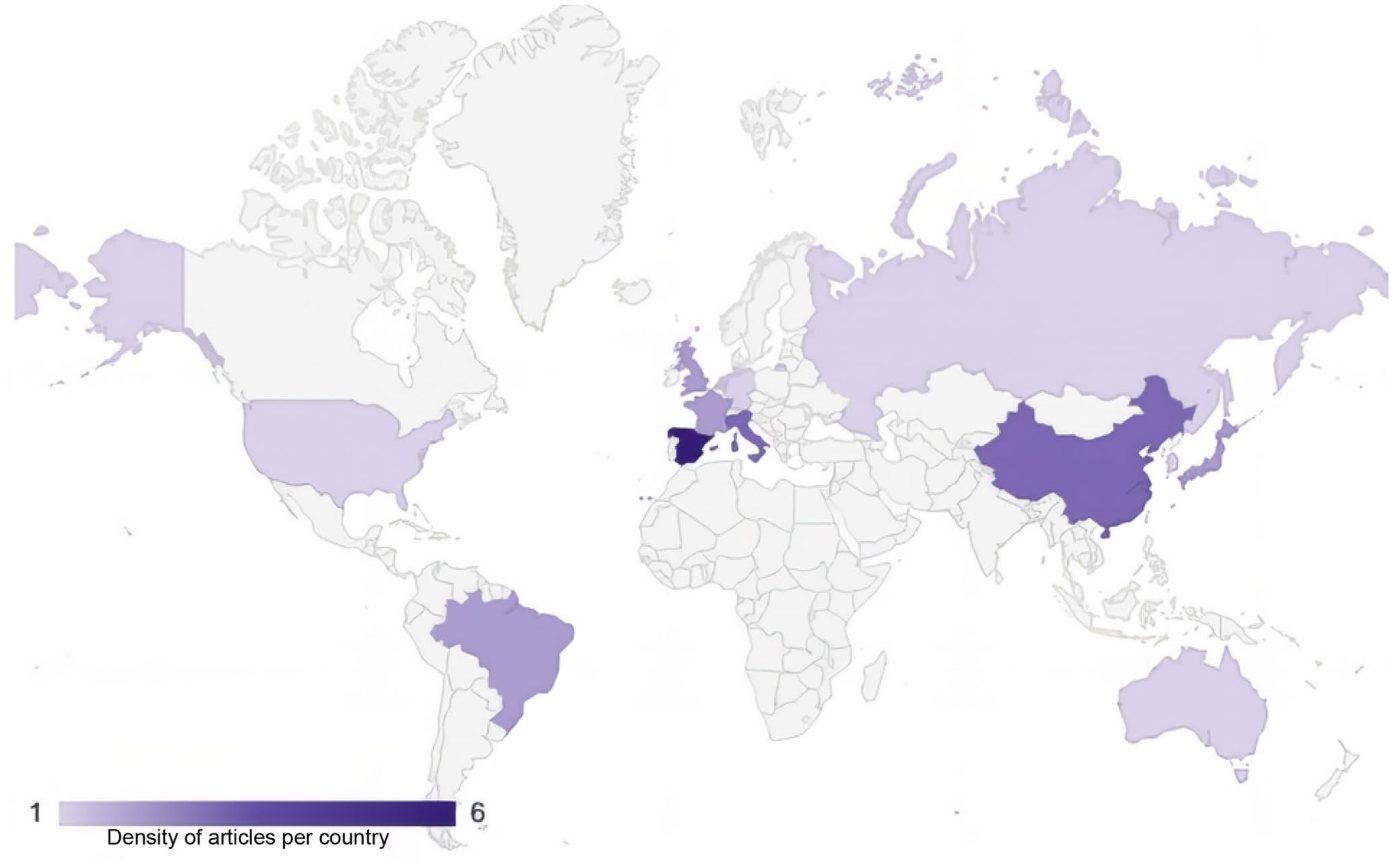
Distribution of articles by country.

The studies included were conducted in 13 countries: Spain (6), Italy (3), China (3), Brazil (2), Japan (2), United Kingdom (2), France (2), Netherlands (1), Russia (1), United States of America (1), South Korea (1), Australia (1) and Germany (1). Although 13 countries are listed, the total number of studies is higher because one study was conducted simultaneously in five European countries (France, Germany, Italy, Spain, and United Kingdom). Another study covered 31 European countries, however, since these were not individually specified, they were not included in the country count (Figure 2).

**FIGURE 3:**
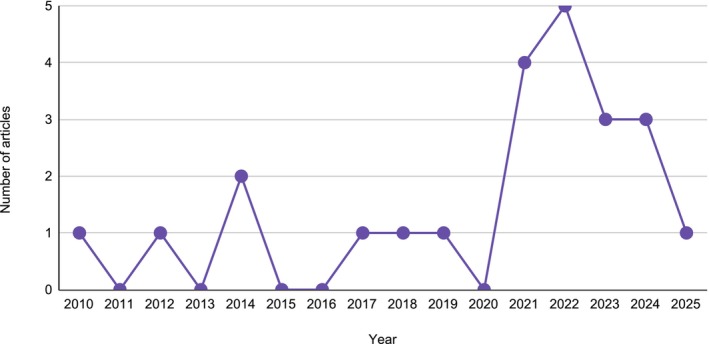
Distribution of articles by Year.

Most of the articles were published in 2022 (approximately 21.7%), followed by 2021, which accounted for 17.4% of the total. In 2023 and 2024, there were three publications each (13%), while 2014 contributed with two publications (8.7%). The remaining years—2010, 2012, 2017, 2018, 2019, and 2025—each had a single publication (4.35%). No publications were recorded in the intervening years (Figure 3).

The included articles were published across a diverse set of journals, each varying in their level of accessibility, indexing, and thematic relevance to RDs. Out of the 16 journals, the majority are indexed in major scientific databases such as PubMed, Scopus, or Web of Science, ensuring baseline visibility within the academic community. Regarding accessibility, over half of the journals operate under a fully open access model, allowing unrestricted public access. Others follow a hybrid model, offering open access for specific articles upon payment of publication fees, with a subscription‐based minority. In terms of thematic focus, only a few journals specialize explicitly in RDs—such as *Orphanet Journal of Rare Diseases* and *Intractable and Rare Diseases Research*—while others, such as *Frontiers in Endocrinology* or *IJERPH*, include RDs as part of a broader scope. One of the included sources, the Brazilian Medical Genetics Congress Annals, although openly available online, is not indexed in major international databases, limiting its reach despite its accessibility. This distribution highlights a moderate degree of alignment between the publication venues and the visibility needs of RD research, with accessibility being relatively strong, but thematic specialization still limited to a small number of journals (Table S3).

The majority of the included articles (52.2%) were cross‐sectional studies. The remaining comprised various study designs, including historical cohort studies (17.4%), qualitative studies (13%), as well as case series/meta‐analysis, reviews, letters to the editor and case–control studies (each 4.35%). Among the 23 studies included, data collection methods varied. Questionnaires administered to patients and/or their families were utilized in 9 studies, while interviews were conducted in 5. One study employed both questionnaires and interviews. Additionally, 6 studies collected data through the review of medical charts or database searches, and 1 relied on literature searches. One study, published as a letter to the editor, did not involve the use of any data collection instrument.

Sample sizes varied widely, ranging from as few as 9 patients with HAE; 13 with MKD; and 25 patients with various RDs, to much larger cohorts, such as 6507 RD patients in one study and 1692 with Acromegaly in another.

The mean time to diagnosis was assessed across articles focusing on specific RDs, revealing considerable variability. The longest reported diagnostic delays were observed in HHT, with a mean of 25.7 years, and HAE, with 23 years. Other notable delays included MKD with 7.1 years, DRD with 5.6 years in a case series and 14.6 years in a systematic review, and SN with a median of 5.4 years. Alpha‐mannosidosis showed a mean diagnostic delay of 4.8 years. Acromegaly was assessed in two studies, with diagnostic delays of 4.4 and 1.4 years. GD had a mean delay of 1.2 years.

**FIGURE 4:**
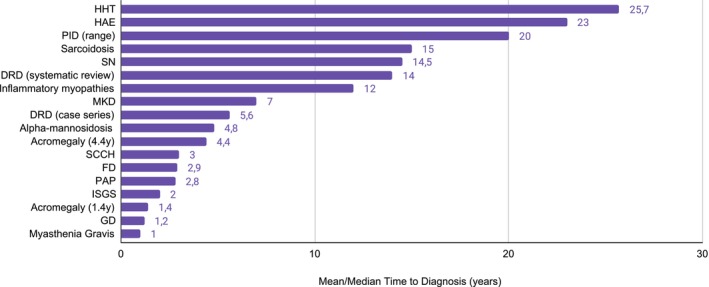
Diagnostic delay by disease (averages).

Some studies reported broader diagnostic ranges rather than exact means. Inflammatory myopathies had delays ranging from 6 months to 12 years; sarcoidosis from 6 months to 15 years; and PID up to 20 years. Myasthenia gravis had an average delay of approximately 1 year. PAP had a mean diagnostic time of 2.8 years, FD of 2.9 years, ISGS a median of 2 years, and SSCH ranged from 1 month to 3 years between the first consultation and diagnosis (Figure 4).

### 3.3 Reasons Attributed to Diagnostic Delay

Overall, the included studies had several common themes, which were grouped and summarized in two comprehensive tables. The first table details the consequences of diagnostic delay for specific RD groups and the corresponding reasons attributed to the delay (Table 2). The second outlines the repercussions for RD patients as a whole, and the broader reasons for late diagnosis (Table 3).

**TABLE 2:** Presentation of the main findings and reasons attributed to the diagnostic delay of the specific group of RDs.

Study	Disease	Main findings	Reasons attributed to the diagnostic delay
39	Hereditary Angioedema (HAE)	HAE is a life‐threatening condition with a mean diagnostic delay of 23 years. During the diagnostic process, patients reported an incessant search to understand the triggers for their symptoms, even altering their diets to identify potential connections. Additionally, multiple hospitalizations and various unnecessary exams were reported, including one case of abdominal surgery. Consequently, more than half of the patients in this study ceased hospital visits, as the tests failed to yield abnormal results. Moreover, several patients received alternative diagnoses for their clinical condition and were prescribed medication accordingly. Some patients attributed their symptoms to psychosomatic factors and blamed themselves	A general lack of clinical suspicion of RDs among healthcare professionals; Limited access to accurate and disease‐specific information at the point of care; Patients often become habituated to their symptoms, leading them to discontinue seeking medical attention.
65	Idiopathic subglottic stenosis (iSGS)	This retrospective review of 124 patients demonstrates that iSGS is a condition with a mean diagnostic delay of 24.5 months Throughout their diagnostic odyssey, many patients were misdiagnosed with various respiratory diseases, predominantly asthma, resulting in unnecessary treatment with inhaled steroids Additionally, the delay in diagnosis resulted in numerous hospitalizations, procedures, and even unnecessary surgeries. Beyond the impact on patients' lives, all of these factors contribute to unnecessary healthcare costs	The intrinsic rarity of the condition; Its tendency to affect otherwise healthy individuals, who may delay seeking medical attention; Initial consultations typically occurring in primary care, where familiarity with iSGS is often limited; Clinical features that closely mimic more common respiratory disorders, leading to misdiagnosis; Infrequent or delayed referral to otolaryngology specialists, who are more likely to recognize the condition; Extended wait times for diagnostic procedures and delays in the communication of test results.
69	Dopa‐Responsive Dystonia (DRD)	This study was conducted in two different ways: a case series study and a systematic review of the literature The case series study was conducted among patients with DRD in a tertiary center, revealing an average delay of 5.6 years. Data showed that many patients were misdiagnosed, leading to unnecessary tests, surgeries, and treatments The systematic literature review focused on understanding the consequences of diagnostic delay in DRD patients. The literature revealed a diagnostic delay of 14.6 years, with later diagnoses being associated with a higher likelihood of incomplete resolution of motor symptoms of the disease	The wide variety of atypical clinical presentations, which complicates early recognition; Patients who underwent surgeries based on an initial misdiagnosis—most commonly cerebral palsy—faced even greater diagnostic delays as a result.
48	Gaucher Disease (GD)	The article focuses on describing healthcare service utilization by patients with GD in China, estimating the associated costs of illness and evaluating the quality of life for both patients and their caregivers. Data shows that the median time to reach an accurate diagnosis among a sample of 49 patients was 1.2 years One consequence of this diagnostic delay was that, due to it and the unavailability and unaffordability of expensive drugs, almost 80% of patients lacked confidence in future treatments Additionally, patients had to visit an average of 3.9 hospitals for diagnosis, exacerbating the financial burden	Limited awareness and understanding of Gaucher disease among healthcare professionals; Wide variability in the disease's clinical manifestations, which can obscure early diagnosis.
25	Acromegaly	Acromegaly is a rare endocrine condition characterized by excessive secretion of Growth Hormone (GH) and Insulin‐like Growth Factor (IGF‐1) In this study, 22.6% of patients experienced delayed diagnosis, leading to a lack of treatment or receiving only symptomatic treatment until the correct diagnosis was made Additionally, these patients were less likely to be referred to other physicians for further evaluation. Furthermore, patients who experienced diagnostic delay presented with a greater number of initial symptoms, exhibited higher GH levels at the time of diagnosis, and were more likely to report musculoskeletal and endocrine‐metabolic conditions other than diabetes at the time of the survey. They also had a higher likelihood of being misdiagnosed	Limited knowledge and awareness of acromegaly among non‐specialist healthcare providers; Ineffective communication between patients and physicians, hindering symptom recognition; Insufficient clinical information and time constraints during consultations, limiting thorough patient evaluation and delaying appropriate referrals.
60	Mevalonate Kinase Deficiency (MKD)	MKD is a rare rheumatological condition that leads to the overproduction of proinflammatory substances Study data showed an average diagnostic delay of 7.1 years, during which patients experienced numerous misdiagnoses, and multiple hospitalizations As the most common symptom is recurring fever, many patients were hospitalized and treated for infections they did not have. Additionally, since the disease can cause relative immunodeficiency, a considerable number of patients required hospitalization to treat serious conditions such as pulmonary or gastrointestinal infections. Some participants were also subjected to iatrogenic exams and other inappropriate treatments	Limited physician knowledge about mevalonate kinase deficiency (MKD); Incomplete or flawed clinical data collection during consultations; Inadequate diagnostic verification and follow‐up; Frequent misdiagnosis as more common conditions, due to the rarity and low recognition of MKD.
6	Pulmonary Alveolar Proteinosis (PAP)	PAP is a RD with a mean time to diagnosis of 34 months. Given that its primary symptoms mimic a wide array of lung conditions, it is frequently misdiagnosed, resulting in patients receiving unnecessary treatments, such as antibiotics Apart from enduring the typical side effects of these treatments, misdiagnosis can lead to delayed diagnosis, consequently postponing appropriate treatment and exacerbating the patient's condition	Nonspecific clinical manifestations that frequently lead to misdiagnosis; Delayed recognition of the correct diagnosis due to the overlap of symptoms with other conditions; Underutilization of disease‐specific radiologic findings, which—if considered—could facilitate earlier identification of pulmonary alveolar proteinosis (PAP).
12	Hereditary Hemorrhagic Telangiectasia (HHT)	Patients with HHT experienced an average diagnostic delay of 25.7 years This delay resulted in postponed treatment, including preventive measures for AVMs. The absence of timely intervention can precipitate severe complications, given the disabling sequelae often associated with AVMs, necessitating costly support and/or pharmacological interventions Moreover, diagnostic delays can impose significant financial burdens due to the irreversible damage inflicted on patients' lives	Limited understanding of HHT among healthcare professionals; Heterogeneous clinical manifestations that vary widely between patients; Symptoms that mimic more common diseases, leading to frequent misdiagnosis; Absence of a reliable biochemical test, making confirmation of the diagnosis more challenging.
9	Sternocostoclavicular Hyperostosis (SCCH)	The study examines the burden of SCCH, a rare, chronic, and inflammatory condition, throughout patients' diagnostic odyssey, with a mean time for a definitive diagnosis ranging from 1 month to 36 years It was found that the social support provided to these patients is inversely related to the time required to obtain the correct diagnosis, indicating a potential decrease in support over the course of the diagnostic process Additionally, the research revealed that many patients experienced significant psychological impacts, with some even exhibiting PTS symptoms due to uncertainty surrounding their correct diagnosis and treatment. Furthermore, the data indicated that nearly one‐fifth of the patients lost confidence in the healthcare system due to diagnostic delays	Lack of knowledge about SCCH among healthcare professionals, as suggested in the literature, contributes to its frequent underdiagnosis—despite participants not explicitly identifying specific reasons for diagnostic delay.
64	Sensory Neuronopathy (SN)	SN is a syndrome that can be idiopathic (primary) or secondary to other primary conditions. Its signs and symptoms vary and can resemble those of other neurological and non‐neurological disorders On average, it took participants 5.4 years to receive a diagnosis. Consequently, SN is frequently misdiagnosed, leading to unnecessary treatments. During their diagnostic journey, patients consulted an average of 4.3 physicians and received a mean of 3.4 incorrect diagnoses This delay caused significant distress due to the prolonged diagnostic process and incorrect treatments, negatively impacting their quality of life, particularly through ataxia and unemployment. Additionally, the identification of possible associated diseases was often delayed	Symptom onset after the age of 40 was associated with longer diagnostic delays compared to earlier onset; SN is rarely considered in the differential diagnosis by general practitioners, reflecting limited awareness and knowledge among physicians.
76	Fabry Disease (FD)	FD is a genetic disorder caused by an enzyme deficiency that leads to the abnormal accumulation of substances in various cells and tissues. The average time between the first symptom and diagnosis was 2.9 years (ranging from 0.3 to 11.6 years) A delay in diagnosing FD can result in patients experiencing multiple symptoms, such as abdominal pain and acroparesthesias, which diminish their quality of life. Additionally, a significant number of patients in this study were misdiagnosed, with chronic arthritis and intermittent labyrinthitis being common diagnoses Moreover, by the time they received the correct diagnosis of FD, four patients in this study had already developed cardiovascular dysfunction and renal disease, secondary comorbidities associated with their genetic condition	Wide variability in clinical presentations and symptoms of Fabry disease, making early recognition difficult; Limited awareness and knowledge of Fabry disease among physicians, contributing to delayed diagnosis.
80	Myasthenia Gravis (MG)	The study found that 27.1% of patients with MG experienced a diagnosis delay of more than 1 year, with a median delay of 183 days and a maximum delay of over 14 years. Those with longer diagnostic delays exhibited greater disease burden and symptom severity, particularly in terms of fatigue, anxiety, and depression. Patients with > 1 year delay had higher levels of fatigue (72.4% vs. 61.3%), and the fatigue was more frequently rated as severe and as having a substantial impact on daily life. They also had higher rates of anxiety (30.5% vs. 17.4%) and depression (21.9% vs. 13.1%) compared to those diagnosed within a year Additionally, self‐reported quality of life (MG‐QoL‐15r scores) was lower in the delayed group (mean 14.4 vs. 12.6). These findings suggest that diagnostic delay in MG is associated with worse clinical outcomes, more comorbidities, and reduced health‐related quality of life	Frequent misattribution of symptoms like fatigue and muscle weakness to common or nonspecific conditions (e.g., chronic fatigue syndrome, hysteria, critical neuropathy); High rate of prior misdiagnosis: among patients with > 1 year delay, 69.2% had at least one incorrect diagnosis, with some receiving two or three; Fluctuating and non‐specific presentation of myasthenia gravis (MG) symptoms, making recognition more difficult; Presence of comorbidities and potential diagnostic bias, particularly affecting women and older adults; Lack of awareness about MG among healthcare professionals; Complex diagnostic pathways involving multiple providers, contributing to prolonged time to accurate diagnosis.

**TABLE 3:** Presentation of the main findings and reasons attributed to the diagnostic delay of the general group of RDs.

Study	Main findings	Reasons attributed to the diagnostic delay
34	This study focused on analyzing the psychological impacts of being diagnosed with a RD and the consequences thereof, depending on whether patients experienced diagnostic delays The results revealed that patients who underwent diagnostic delays required psychological support more frequently during their search for a diagnosis. Additionally, these patients reported difficulties in their social lives, finding it challenging to explain their symptoms to others or justify absences for medical reasons, whether in occupational or educational settings. Loss of opportunities, both in personal and corporate life, was also cited. Complaints regarding the loss of independence due to disease progression during the diagnostic delay were also common	The participants did not specify the reasons they believed contributed to their diagnostic delays; however, the literature reviewed by the authors identified several key determinants: Need to travel long distances to consult with specialists; Patients often consult multiple physicians before receiving a diagnosis; Diagnostic delays tend to be longer for nervous system disorders; Limited scientific knowledge and availability of diagnostic tests; High heterogeneity among rare diseases, complicating timely recognition and diagnosis.
5	The article was based on a cross‐sectional study that examines the effects of misdiagnosis and delayed diagnosis on patients' trust in physicians The data revealed that patients with a time to definitive diagnosis exceeding 1 year had lower trust scores than those diagnosed within that timeframe. Additionally, although it was found that misdiagnosis alone had a lower influence on trust scores, patients who experienced misdiagnosis and had to wait more than 1 year for their final diagnosis had the lowest trust scores in physicians	Geographic barriers, with most patients living far from specialized reference centers and needing to travel long distances for care; Patient‐related factors, such as physical disabilities, which can hinder timely access to diagnostic services
32	The primary focus of this research was to assess the determinants of delayed diagnosis and the diagnostic journey of rare diseases Although the consequences of such delays were not the main objective, pertinent data were also obtained. It was observed that many patients with delayed diagnoses had to seek specialists outside their Autonomous Region. Furthermore, the frequency of medical tests, hospitalizations, and surgical interventions was higher among patients experiencing diagnostic delays. All these factors contribute to the financial burden experienced by patients and their families	Higher likelihood of diagnostic delay when patients first sought help from primary healthcare providers; Increased risk of delay when travel to reference centers or hospitals in other regions was required; Consulting more than 10 specialists was associated with prolonged diagnostic timelines; A greater number of tests and surgical interventions correlated with longer time to diagnosis; RDs affecting the nervous system were more commonly associated with diagnostic delays.
33	This study revealed that patients who experienced diagnostic delays longer than 1 year utilized medications and other treatments to a greater extent and exhibited a higher demand for healthcare products, which were also more commonly unavailable for this group. Consequently, pharmaceutical expenses were higher among patients with diagnostic delays, adding financial burden to the list of its consequences Furthermore, while families primarily attributed emotional impacts to the disease itself rather than the delay in diagnosis, it is conceivable that the latter partially contributes to the social and psychological repercussions	The article didn't approach the reasons attributed to the diagnostic delay
74	The aim of this study was to analyze the impact of diagnostic delay and associated psychosocial needs in patients with RDs. The authors conducted interviews with RD patients and compared the responses of those without diagnostic delays to those experiencing delays of more than 5 years in obtaining a correct diagnosis. They discovered that patients with delayed diagnoses faced heightened uncertainty and anxiety during the pre‐diagnosis period compared to those diagnosed within a year, leading to significant emotional distress that impacted their family relationships. The group with delayed diagnoses emphasized the lack of psychological support and expressed concerns about their quality of life due to uncertainty regarding disease progression	Asymmetric and paternalistic physician–patient relationships, which can hinder effective communication and contribute to diagnostic delays; Limited understanding of rare diseases among healthcare professionals, prolonging the diagnostic process.
75	The authors analyzed data from six reference medical centers in Galicia, encompassing a total of 324 patients, and found an average delay in the diagnostic process of rare diseases of 4.8 years. Additionally, about one third of the patients had to wait for 5 or more years to receive their correct diagnosis. Patients with a diagnostic delay greater than 5 years scored lower on the Barthel Dependency Scale, indicating less independence in their daily tasks compared to patients with earlier diagnoses. Data also revealed that this group had a 1.75 times higher risk of utilizing more healthcare resources. The age at diagnosis, number of annual outpatient visits, and number of medical specialists consulted were significantly higher in the group with longer diagnostic delays. Furthermore, the authors noted that patients with delayed diagnoses of genetic rare conditions are deprived of appropriate genetic counseling and proper family planning	Lack of scientific knowledge or clinical experience with rare diseases among physicians, identified as the primary factor; Absence of specialized reference centers and difficulty in referring patients to them; Limited access to genomic testing, which can hinder timely diagnosis.
43	This article is a letter to the editor describing the author's perception of living with a rare disease and experiencing diagnostic delays; the author itself waited 17 years for a proper diagnosis It is mentioned that the delay in diagnosis and the psychological condition of a patient often interact in a two‐way manner, with one exacerbating the other. Healthcare professionals frequently dismiss patients' conditions, attributing symptoms to the patient's psychiatric condition, prolonging the delay. This process leaves patients feeling invalidated, mistrusted, and hopeless, further exacerbating their mental illness. Diagnostic delays prevent patients from receiving proper treatment and may contribute to disease progression The prolonged duration of this process may also lead patients' families to become accustomed to their symptoms, leading them to believe the patient is not actually ill, further invalidating the patient and causing distress	Physicians' failure to recognize symptoms, often misattributing them to psychosomatic or mental health conditions; Invalidation of patients' complaints, which discourages further investigation and delays diagnosis; High variability in symptom presentation, complicating the identification of the underlying condition.
30	The article consists of a review of ongoing international initiatives aimed at reducing the late diagnosis of rare diseases and its ethical and social impacts The authors first address the consequences of delayed diagnosis and present strategies to mitigate this issue. It is highlighted that diagnostic delay prevents patients from receiving specialized healthcare promptly, which may lead to disease progression. The absence of a diagnosis forces patients and their families to rely solely on their own resources, as accessing social services becomes challenging without a formal diagnosis. Furthermore, delayed diagnosis can result in unnecessary appointments and procedures. Psychological and social consequences, such as a sense of isolation and implications for reproductive choices, are also significant, as the lack of a proper diagnosis hinders patients from making informed decisions	Lack of knowledge about RDs among physicians; Wide variety of signs and symptoms, often presenting atypically; Miscommunication between doctors and patients, hindering accurate assessment; Involvement of non‐genetic risk factors that are poorly understood or not clearly identified.
82	This large‐scale European survey found that the average time to diagnosis (TDT) for people living with a rare disease was 4.7 years, with 25% of patients waiting more than 5 years and 48% waiting over 1 year for a confirmed diagnosis. The consequences of misdiagnosis and delay were substantial: 68% of affected respondents reported delayed access to appropriate care, 59% were prevented from accessing proper treatment, and 52% received inappropriate interventions. Additionally, more than half of the participants reported a worsening of symptoms during this period. Despite some improvements in disease understanding and access to treatment post‐diagnosis, social life was reported to worsen by 53% of respondents, and access to financial and social support services often remained unchanged. These results highlight the broad medical, emotional, and social toll of delayed diagnosis in the rare disease community	System‐level contributors: Health system delays accounted for 90% of the total diagnostic timeline, with a median of 4.3 years from first medical contact to diagnosis; High rate of misdiagnosis (reported by 73% of patients); Consultations with multiple healthcare professionals, especially eight or more; Reluctance among providers to order genetic testing; Limited referrals to Centres of Expertise; Regional disparities, with longer delays reported in Western and Northern Europe compared to Eastern and Southern regions. Patient‐level contributors: Higher likelihood of delay among women and younger individuals at symptom onset; Unmet psychological and financial support needs; Presence of a family history of the same rare disease, possibly leading to normalization of symptoms or delayed pursuit of formal diagnosis.

The most consistently reported factor with diagnostic delays in RDs was the lack of knowledge and experience among healthcare professionals. This was frequently associated with difficulties in recognizing symptoms as potentially indicative of specific RDs, as well as a general lack of clinical suspicion. In addition, the phenotypic heterogeneity and clinical variability of RDs were also commonly described as barriers, as diverse presentations can complicate timely recognition.

Another significant issue was the power imbalance between physicians and patients. This was often linked to the invalidation of patients' symptoms, with many individuals being misdiagnosed with psychosomatic disorders. Such dynamics disrupted communication and contributed to delays in diagnosis. Moreover, insufficient time for a thorough investigation of the patients' medical history was also cited as a contributing factor.

Diagnostic‐related factors, such as long wait times for test results and lack of access to genetic testing, were also described across the included studies. Other recurring barriers included limited access to specialists, fragmented care and inadequate referral pathways. RDs with neurological manifestations, diagnostic overlap with other conditions, and discontinuation of medical follow up were likewise cited as contributing factors.

### 3.4 Consequences of Diagnostic Delay

Misdiagnoses and inappropriate treatments emerged as central and repeatedly described consequences of diagnostic delay. These diagnostic errors were associated with suboptimal management strategies and subsequent clinical deterioration of patients. Beyond these misdirected interventions, broader clinical impacts were also widely documented. Hospitalizations, surgeries and frequent consultations with various specialists reflected the complexity and severity of undiagnosed conditions and were described across most of the reviewed studies. In many cases, patients underwent procedures that were ultimately unnecessary, further exposing them to risks and delays in receiving appropriate care.

In close connection with these clinical challenges, psychological impacts were another recurring theme. The extended uncertainty and repeated diagnostic failures intensified stress, anxiety, and depression levels, severely affecting patients' overall well‐being. Many noted the absence of psychological support during their diagnostic journey, emphasizing the need for enhanced emotional and mental health care. The diagnostic odyssey was frequently described as emotionally distressing, with patients reporting feelings of invalidation and hopelessness, which, when compounded by ongoing physical suffering, led to a marked decline in quality of life, as reported across the publications.

These emotional and physical effects were often described as being amplified by disease progression, further illustrating the clinical dangers of delayed diagnosis. Without timely intervention, many conditions progress to more severe stages, making treatment more complex and the prognosis less favorable.

The consequences extend beyond health itself. Financial burdens were frequently reported, encompassing not only direct costs, such as medical care and hospitalizations, but also indirect costs related to caregiving, travel and accommodation for medical appointments. The prolonged diagnostic process often contributes to increased healthcare expenditures as patients undergo repeated testing, consult multiple specialists and, in some cases, receive unnecessary surgeries and procedures before receiving an accurate diagnosis, thereby significantly increasing expenses and imposing long‐term financial strain.

Social relationships are not spared from the toll of delayed diagnosis. Studies consistently described social isolation and relational stress that often accompany chronic illness. In addition to increased social withdrawal, one publication noted that prolonged pre‐diagnosis periods could lead family members to perceive the patient as exaggerating or not genuinely ill, resulting in emotional distress and strained relationships (Nunn 2017). Another study found an inverse relationship between the time required to obtain a diagnosis and the level of social support received, suggesting that the longer the diagnostic process lasts, the more patients tend to lose support from their social networks (Van der Kloot et al. 2010). Collectively, these findings reveal the substantial psychosocial toll of diagnostic delays in RDs, affecting both patients and their immediate social circles.

Finally, a strained doctor‐patient relationship was also identified as a significant repercussion. This finding suggests that, as disease progresses without a diagnosis, patients may lose trust in their healthcare providers. A Japanese study (Tanaka and Shimaoka 2023) emphasized this dynamic, showing that extended diagnostic journeys can place significant strain on communication and trust between patients and clinicians, further exacerbating the challenges faced by patients with RDs.

### 3.5 Addressing the Articles Limitations

This review also examined the limitations reported across the included studies (Table S4), as these factors directly affect the strength and generalizability of the findings. A common issue identified was participant bias, particularly in studies involving older individuals or those with more severe disease manifestations. Such bias may skew results and lead to underrepresentation of younger or less severely affected populations.

Selection bias was acknowledged in nearly half of the studies particularly in those conducted at specialized reference centers. While some authors recognized this as a limitation, others argued that their findings nonetheless held broader relevance.

Recall bias was another recurring concern, arising from the reliance on participants' memory to report symptom onset or healthcare utilization. This may introduce potential inaccuracies, especially in retrospective studies. Small sample sizes were another recurrent limitation, restricting both the statistical power and the capacity to conduct detailed subgroup analyses.

Additional reported limitations included incomplete data and disease representation which limit the generalizability of findings to the broader rare disease population. The COVID‐19 pandemic also introduced challenges in at least one study, such as methodological changes, digital exclusion, and gender disparities in survey responses. Less commonly noted concerns included temporal bias, which reflects difficulties in assessing changes in diagnostic timelines over extended periods due to evolving clinical practices, updated disease definitions, and advances in genetic testing.

## 4 Discussion

The reviewed studies covered a range of RDs, with nine broadly addressing diagnostic delays across RDs and 15 focusing on specific individual diseases. Due to their low prevalence, these conditions pose substantial challenges for timely diagnosis and treatment initiation and once diagnosed, they often require long‐term and expensive treatments, raising concerns among national governments (NHS England n.d.).

To further explore the epidemiological dimension of RDs, prevalence data were sourced from two major reference websites on RDs: Orphanet and NORD (Orphanet n.d.; National Organization for Rare Disorders 2015). Discrepancies were found between the prevalence figures reported by each source. This is likely due to differences in their geographic focus—Orphanet provides a global perspective and includes some region‐specific epidemiological data, whereas NORD focuses primarily on data from the US. An additional database: the Genetic and Rare Diseases Information Center (GARD) (Tanaka and Shimaoka 2023), was also consulted; however, prevalence data was not available. This highlights the overall scarcity of data on RDs and underscores the challenges in advancing policy in this area.

The high volume of studies from European countries can be largely attributed to substantial funding from the European Union's research and innovation framework programs, such as Horizon 2020 and the Seventh Framework Programme (European Commission 2024). In addition, collaborative networks like EURORDIS—which advocates for people living with RDs—and the European Reference Networks (ERNs)—networks of specialized centers across Europe—play a crucial role in supporting research efforts (EURORDIS n.d.; Public Health 2025). These initiatives provide structured platforms for knowledge‐sharing, resource allocation, and the exchange of best practices, fostering an environment conducive to research and publication.

Within this European context, Spain has emerged as a key contributor to RD research at the national level. The development of the Rare Diseases Strategy of the Spanish National Health System in 2006 led to the establishment of the Spanish Rare Diseases Patient Registry Research Network (Ministry of Health and Social Policy 2009; Vicente et al. 2019), which has since supported national data collection and policy development. A notable example of Spain's leadership is the ENSERio study (2016–2017), which examined the experiences of families affected by RDs. The study revealed that diagnostic delays were primarily caused by a lack of awareness and knowledge among healthcare professionals, and highlighted the significant psychological, physical, and emotional impacts of such delays on patients and their families (Solves Almela et al. 2018). According to the findings, 7.6% of patients had a non‐definitive diagnosis, while 3.2% remained undiagnosed.

Other European countries, particularly France and Germany, also have strong policies and frameworks for supporting RD patients, despite contributing fewer publications related to this specific research theme. France adopted its First National Plan for Rare Diseases in 2004 (French National Plan for Rare Diseases 2005–2008 2004), which became a model for other European Union member states to develop their own national strategies. Both France and Germany benefit from centralized national funding, broad access to treatment, research initiatives, and coordinated networks, all of which contribute to their robust RD infrastructures (Dharssi et al. 2017).

Beyond Europe, Asian countries such as China and Japan have made significant advancements in RD policy through structured national strategies and dedicated healthcare efforts. In China, a major milestone was the release of the first National List of Rare Diseases in 2018, listing 121 conditions, followed by the establishment of diagnostic and treatment networks, expert panels, and alliances to strengthen national coordination (Ying et al. 2021). The country has also implemented regulatory incentives for orphan drug development and included some RD treatments in its national insurance system (Ying et al. 2021). Despite these achievements, China continues to face important challenges, including uneven distribution of healthcare resources, limited awareness among healthcare professionals, fragmented data systems, and a lack of unified diagnostic protocols across institutions (Ying et al. 2021). Japan, in turn, has demonstrated long‐standing commitment to RDs through its Nanbyo policy, which has supported patients with designated intractable diseases since the 1970s (Tang and Makuuchi 2012). Its centralized health insurance system facilitates access to care and early implementation of orphan drug legislation (Tang and Makuuchi 2012). However, the narrower definition of RDs adopted in Japan limits the number of recognized conditions and, consequently, the scope of coverage and research efforts (Tang and Makuuchi 2012). These differences underscore how national health systems and policy frameworks shape the scope and direction of RD research and care.

In Brazil, the policy for comprehensive care for people with rare diseases, launched in 2014, within the Brazilian Unified Health System (SUS), laid the foundation for national RD infrastructure (Ministério dos Direitos Humanos e da Cidadania n.d.). To support its implementation, the Brazilian Rare Diseases Network was developed, comprising over 40 institutions, including 18 public university hospitals, 17 rare disease reference services, and 5 newborn screening reference services (Félix et al. 2022; Iriart et al. 2019). These institutions provide outpatient consultations, genetic testing, and genetic counseling for patients with specific RDs, all under public funding. The network also serves as the basis for the national research protocol described in 2022 by Félix et al., which outlines a structured, two‐phase epidemiological study, using standardized tools to map the clinical, diagnostic, and operational aspects of RD care in Brazil (Félix et al. 2022). Despite this framework, disparities remain in access and geographic coverage, particularly in underserved or remote areas.

To evaluate how this network has been functioning in practice, the Brazilian Rare Diseases Network (RARAS) national survey analyzed data from 34 participating centers, offering a comprehensive picture of the Brazilian RD landscape. Among 12,530 patients surveyed, 63.2% had a confirmed diagnosis, 17.3% remained undiagnosed, and the average diagnostic delay was 5.4 years, aligning with global averages (Mello et al. 2024). Importantly, the Brazilian Unified Health System (SUS) was the primary funder for both diagnosis (84.2%) and treatment (86.7%), highlighting the critical role of the public health system. However, persistent challenges remain. As Iriart et al. (Iriart et al. 2019) emphasized, many patients face long journeys even after reaching specialized centers, due to bureaucratic hurdles, insufficient provider training, and limited access to high‐cost medications.

The RARAS findings further reinforced these difficulties by identifying that the main reasons for diagnostic delay in Brazil are the scarcity of specialized centers, the unequal geographic distribution of services, and the shortage of trained specialists, especially in underserved regions such as the North and Northeast. These systemic barriers not only prolong the diagnostic odyssey but also exacerbate inequalities in access to care. Expanding the coverage of reference centers, investing in professional training, and improving regional equity are therefore crucial steps to reduce diagnostic delays in the Brazilian context (Mello et al. 2024).

As for publication trends, there has been a clear rise in recent years: 17 out of 23 reviewed articles were published in the last 5 years. This surge reflects increased awareness and recognition of the importance of timely RD diagnosis. Earlier years, like 2010, had minimal output (only one publication), underscoring the recent intensification of research interest. This trend aligns with the clinical and economic benefits of early diagnosis, which improves outcomes and helps reduce disparities (Chung et al. 2022). Collectively, the data indicate a broader global push toward reducing diagnostic delays and investing in early intervention strategies.

Regarding journal accessibility, thematic specialization and equity in scientific publishing warrant further discussion. Although the journals included in this review were largely accessible—many operating under open access (OA) models and indexed in major databases—only a minority were thematically specialized in RDs. This scarcity of dedicated publication venues reflects broader challenges affecting the visibility of RD research. Wang et al. emphasize that RDs remain inconsistently categorized and underrepresented, and propose a globally endorsed operational definition to improve recognition and visibility (Wang et al. 2024). Establishing a shared conceptual framework is essential not only for guiding policy, but also for shaping research agendas. Likewise, Kilgallon et al. highlight persistent inequities in scientific publishing, especially for researchers in low‐ and middle‐income countries, who face high article processing charges and limited OA options (Kilgallon et al. 2023). These disparities are particularly critical in RD research, which depends on international collaboration and often lacks funding. Thus, despite increased accessibility, limited specialization and financial barriers may hinder the equitable dissemination of knowledge (Kilgallon et al. 2023).

Across studies, the main reasons for diagnostic delays in RDs were consistent, with the most frequently cited factors being healthcare professionals' lack of knowledge and experience, and the heterogeneous and complex nature of these conditions. Additional barriers included power imbalances in physician‐patient relationships, delays in test results, and limited access to specialized care, highlighting the multilayered challenges that patients often face before receiving a diagnosis.

The impacts of these delays were similarly extensive. Psychological effects were consistently described across studies, while clinical consequences included frequent hospitalizations, surgeries, and unnecessary procedures, contributing to disease progression and treatment complications in a substantial proportion of cases. Economic burdens also emerged as a recurring theme, with prolonged diagnostic journeys often leading to rising healthcare costs and ineffective initial treatments. Additionally, social repercussions, such as strained relationships and isolation, further compounded the overall burden on patients and their families.

These findings are reinforced by results from large‐scale international surveys not included in our review. For instance, an EURORDIS study found that 25% of patients waited between 5 and 30 years for a genetic diagnosis, and 40% received multiple incorrect diagnoses, often leading to ineffective or harmful treatments (EURORDIS 2024b). Another study reported that patients without a confirmed diagnosis faced an average delay of 10 years, with 12% waiting over 20 years. Among these, 90% believed that healthcare providers outside hospital settings lacked adequate knowledge, correlating with longer delays (4.5 vs. 1.5 years). Additionally, 57% underwent inappropriate tests or treatments, and 72% reported poor coordination of care, particularly between hospital and primary care providers. These external findings not only reinforce the psychological, clinical, and systemic consequences identified in our review but also highlight the global urgency of reducing diagnostic delays and improving care coordination for RD patients.

Finally, some limitations of this scoping review should be acknowledged. First, the inclusion criteria restricted articles to those published in Portuguese, English, and Spanish, potentially excluding relevant studies in other languages. This language limitation may have introduced geographic and cultural biases, as research conducted in non‐included regions could offer alternative perspectives or highlight challenges not captured in our sample. Second, some studies had small sample sizes, limiting the possibility of subgroup analyses and reducing the generalizability of findings. Additional issues included incomplete data and the underrepresentation of specific RDs, which may affect the applicability of results to the broader RD population.

Another important limitation relates to our definition of diagnostic delay, which was set at a minimum of 1 year. This threshold was chosen to focus on more substantial delays likely to have meaningful clinical and psychological impacts. However, this approach may have excluded studies examining shorter delays, which could still be significant—particularly for conditions where early intervention is critical. While the one‐year criterion allowed us to capture more pronounced cases, it may have limited the scope of our analysis. Future research including a broader range of delay durations would contribute to a more comprehensive understanding of diagnostic trajectories and their effects across different RDs.

## 5 Conclusion

This scoping review underscores the pressing need for heightened awareness, improved training, and enhanced resources to effectively address diagnostic delays in RDs. Geographic and temporal trends highlight active research areas and an increasing acknowledgment of this issue, while the varied study designs and methodologies underscore the complexity of the problem.

Diagnostic delays in RDs have far‐reaching impacts, affecting patients both clinically and psychosocially, as well as their families. Clinically, prolonged diagnostic journeys can worsen disease progression, leading to severe health complications and reduced treatment effectiveness. Patients often undergo unnecessary procedures and incorrect treatments, further complicating their condition. Psychosocially, the uncertainty and frustration of lacking a definitive diagnosis cause significant emotional distress, including anxiety and depression. Family members, who often take on caregiving roles, experience heightened stress and burnout, which can strain family dynamics and relationships. Social isolation and stigma are common as patients withdraw to avoid judgment or misunderstanding, while financial burdens from extensive medical consultations and tests add to the stress. Overall, the quality of life and daily functioning of patients deteriorate without a clear diagnosis and appropriate treatment. Addressing both clinical and psychosocial needs through effective coping mechanisms and robust support systems, such as mental health services and patient advocacy groups, is essential. This comprehensive approach can significantly improve the well‐being of patients and their families, underscoring the critical importance of timely and accurate diagnoses in managing RDs.

Although this review did not directly investigate genetic counseling, its findings highlight areas that may demand professional abilities. Because most RDs are genetic, reducing diagnostic delays can facilitate earlier referral to genetic counseling, requiring well‐trained professionals to better support patients and families with evidence‐based information, psychosocial guidance, and planning for future care needs. Our synthesis therefore offers insights that can be translated into practical improvements and educational development within the field of genetic counseling.

To advance our understanding of diagnostic delays and their consequences in RD, future research should also consider varying delay thresholds to capture a broader spectrum of diagnostic challenges and their impacts on patient outcomes.

Enhanced international collaboration, inclusive research approaches, and targeted interventions are key to mitigating the impact of diagnostic delays and improving outcomes for RD patients globally. This review shed light on how researchers, healthcare providers, and policymakers can effectively collaborate in developing new strategies for early diagnosis and better management of RDs.

## Author Contributions

L.R.B. and L.F.V. were responsible for the screening procedure and the selection of the texts, as well as for the writing of this protocol and the original article. F.S. and D.S.G.C. assisted in the screening procedure by removing duplicate articles. F.S. was also responsible for all the figures and spreadsheets used, and for verifying all bibliographic references. D.S.G.C., I.G. and C.M.‐M. reviewed the entire text and endorsed the review. D.S.G.C. also collected methodological data. C.M.‐M. was responsible for coordination and supervision.

## Disclosure

No AI tool is generated.

## Ethics Statement

The authors have nothing to report.

## Consent

The authors have nothing to report.

## Conflicts of Interest

The authors declare no conflicts of interest.

## Supporting information


**Table S1:** Websites searched for gray literature.

**Table S2:** RDs prevalences.

**Table S3:** Journals characteristics.

**Table S4:** Presentation of the studies limitations.

## Data Availability

The data that support the findings of this study are available from the corresponding author, FS, upon request.
