# Supplementary material for: Repercussions of Diagnostic Delay in Rare Diseases
Source: J Genet Couns. 2026 Jul 17;35(4):e70258. doi: 10.1002/jgc4.70258 (PMC13379505; doi:10.1002/jgc4.70258)
Supplement: Supplementary file 1 — Table S1: Websites searched for gray literature. [file JGC4-35-0-s004.docx]

| **Supplementary Table S1** Websites searched for grey literature | |
| --- | --- |
| **Non-Governmental websites** | **Governmental websites** |
| Resource Development (UK) (GOOD DIAGNOSIS, nd) | NHS England (NHS England. n.d) |
| EURORDIS (EURORDIS. 2024c) | National Center of Advancing Translational Science (National Center for Advancing Translational Sciences. n.d.) |
| Brazilian Genetic Medical Congress (CBGM) annals (SBGM. n.d) | Brazilian Department of Health (BVSMS. 2014) |
|  | RARAS network (RARAS, nd) |

References

BVSMS. 2014. “DIRETRIZES PARA ATENÇÃO INTEGRAL ÀS

PESSOAS COM DOENÇAS RARAS NO SISTEMA ÚNICO DE SAÚDE

- SUS Portaria GM/MS no 199 de 30/01/2014(*) Brasília -DF 2014.”

https:// bvsms. saude.gov.br/ bvs/publicacoes/diret rizes_ atenc ao_ integ

ral_pessoa_doencas_ raras_ SUS.pdf.

EURORDIS. 2024c. “Major Survey Reveals Lengthy Diagnostic Delays

for Rare Disease Patients.” https://www.eurordis.org/survey-reveals-

lengthy- diagnostic- delays/.

GOOD DIAGNOSIS. n.d. “Improving the Experiences of Diagnosis for

People with Rare Conditions.” https://geneticalliance.org.uk/wp- conte

nt/uploa ds/2024/01/ Rare-Disea se- UK- Good-Diagnosis- Report-2022-

Final.pdf.

National Center for Advancing Translational Sciences. n.d. “More

Treatments for All People More Quickly.” <https://ncats.nih.gov/>.

NHS England. n.d. “Implementation Plan for the UK Strategy for Rare

Diseases.” https://www.england.nhs.uk/wp- content/uploads/2018/01/

implementation-plan-uk-strategy-for-rare- diseases.pdf.

RARAS. n.d. “Rede Nacional de Doenças Raras.” <https://raras.org.br/>.

SBGM. n.d. “Sociedade Brasileira de Genética Médica.” https://www.

sbgm.org.br/anais- do- congresso.aspx
