# Supplementary material for: Repercussions of Diagnostic Delay in Rare Diseases
Source: J Genet Couns. 2026 Jul 17;35(4):e70258. doi: 10.1002/jgc4.70258 (PMC13379505; doi:10.1002/jgc4.70258)
Supplement: Supplementary file 2 — Table S2: RDs prevalences. [file JGC4-35-0-s001.docx]

**Supplementary Table S2: RDs prevalences**

| Disease | Orphanet | NORD (USA) |
| --- | --- | --- |
| Alpha-mannosidosis | 1-9 per 1,000,000 | 1-5 per 100,000 |
| Inclusion body myositis | 1-9 per 1,000,000 | 10-112 per 1,000,000 |
| Polymyositis | 1-9 per 1,000,000 |  |
| Dermatomyositis | 1-9 per 1,000,000 | 3 per 1,000,000 |
| Primary immune deficiencies |  | Described by subtype |
| Sarcoidosis | 1-5 per 10,000 | 60 per 100,000 |
| Myasthenia gravis | 1-9 per 100,000 | 14-40 per 100,000 |
| Hereditary angioedema (HAE) | 1-9 per 100,000 | 1 per 50,000 |
| Idiopathic subglotic stenosis (ISGS) |  |  |
| Dopa-responsive dystonia | 1-9 per 1,000,000 |  |
| Gaucher disease | 1-9 per 100,000 | 1 per 50,000 to 100,000 |
| Acromegaly | 1-9 per 100,000 | 50-70 per 1,000,000 |
| Mevalonate kinase deficiency (MKD) |  |  |
| Pulmonary alveolar proteinosis (PAP) | 1 per 1,000,000 |  |
| Hereditary hemorrhagic telangiectasia (HHT) | 1-5 per 100,000 | 1 per 5,000 |
| Sternocostoclavicular hyperostosis |  |  |
| Non-paraneoplastic sensory neuropathies |  |  |
| Fabry disease | 1-5 per 10,000 | 1 per 40,000 |
