# Supplementary material for: Repercussions of Diagnostic Delay in Rare Diseases
Source: J Genet Couns. 2026 Jul 17;35(4):e70258. doi: 10.1002/jgc4.70258 (PMC13379505; doi:10.1002/jgc4.70258)
Supplement: Supplementary file 3 — Table S3: Journals characteristics. [file JGC4-35-0-s002.docx]

**Supplementary Table S3: Journals Characteristics**

| Reference | Journal | Articles | Indexed (Pubmed/Scopus/WoS) | Rare Disease Focus | Open Access Policy |
| --- | --- | --- | --- | --- | --- |
| Benito-Lozano et al. 2023, Isono et al. 2022 | PlOS One | 2 | Yes | No | Yes (Fully OA) |
| Tanaka et al. 2023 | Intractable and rare Diseases Research | 1 | Yes | Yes | Yes (Fully OA) |
| Benito-Lozano et al. 2022, Gimenez-Lozano et al 2022, Gainotti et al. 2018 | IJERPH | 3 | Yes | Partially | Yes (Fully OA) |
| Berges et al. 2021 | Laryngoscope | 1 | Yes | No | Hybrid (Optional OA) |
| Rivera Gallego et al. 2021 | Galicia Clin | 1 | Yes | No | Yes (Fully OA) |
| Páramo-Rodriguez et al. 2021 | Gaceta Sanitaria (Barc., Ed.impr.) | 1 | Yes | Yes | Yes (Fully OA) |
| Kim et al. 2021 | Brain & Development | 1 | Yes | No | Hybrid (Optional OA) |
| Qi et al. 2021, Van der Kloot et al. 2010, Pierucci et al. 2012, Phillips et al. 2024 | Orphanet Journal of Rare Diseases | 4 | Yes | Yes | Yes (Fully OA) |
| Wang et al. 2021, Bai et al. 2025 | Frontiers in Endocrinology | 2 | Yes | No | Yes (Fully OA) |
| Berody et al. 2015 | Joint bone Spine | 1 | Yes | No | Hybrid (Optional OA) |
| Ilkovich et al. 2014 | Annals of Clinical and Laboratory Science | 1 | Yes | No | No (Subscription) |
| Van der Kloot et al. 2010 | Health and Quality of Life Outcomes | 1 | Yes | No | Yes (Fully OA) |
| Rolim et al. 2019 | Arq. Neuropsiquiatr | 1 | Yes | No | Yes (Fully OA) |
| Tanaca et al. 2018 | Brazilian Medical Genetics Congress Annals | 1 | No | Yes | Yes (Open, not indexed) |
| Faye et al. 2018 | European Journal of Human Genetics | 1 | Yes | No | Hybrid (Optional OA) |
| Cortés-Vicente et al. 2024 | Annals of Clinical and Translational Neurology | 1 | Yes | No | Yes (Fully OA) |

References:

Benito-Lozano, J., G. Arias-Merino, M. Gómez-Martínez, et al. 2022. “Diagnostic Process in Rare Diseases: Determinants Associated With Diagnostic Delay.” International Journal of Environmental Research and Public Health 19, no. 11: 6456.

Benito-Lozano, J., G. Arias-Merino, M. Gómez-Martínez, et al. 2023. “Psychosocial Impact at the Time of a Rare Disease Diagnosis.” PLoSOne 18, no. 7: e0288875.

Berges, A. J., I. A. Lina, L. Chen, R. Ospino, R. Davis, and A. T. Hillel. 2021. “Delayed Diagnosis of Idiopathic Subglottic Stenosis.” Laryngoscope 132, no. 2: 413–418.

Berody, S., C. Galeotti, I. Koné-Paut, and M. Piram. 2015. “A Restrospective Survey of Patients's Journey Before the Diagnosis of Mevalonate Kinase Deficiency.” Joint, Bone, Spine 82, no. 4: 240–244

Cortés-Vicente, E., A. J. Borsi, C. Gary, et al. 2024. “The Impact of Diagnosis Delay on European Patients With Generalised Myasthenia Gravis. Annals of Clinical and Translational.” Neurology 11: 2254–2267.

Faye, F., C. Crocione, R. Anido de Peña, et al. 2024. “Time to Diagnosis and Determinants of Diagnostic Delays of People Living With a Rare Disease: Results of a Rare Barometer Retrospective Patient Survey.” European Journal of Human Genetics 32: 1–11.

Gainotti, S., D. Mascalzoni, V. Bros-Facer, et al. 2018. “Meeting Patients' Right to the Correct Diagnosis: Ongoing International Initiatives on Undiagnosed Rare Diseases and Ethical and Social Issues.” International Journal of Environmental Research and Public Health 15, no. 10: 2072

Gimenez-Lozano, C., L. Páramo-Rodríguez, C. Cavero- Carbonell, et al. 2022. “Rare Diseases: Needs and Impact for Patients and Families: A Cross- Sectional Study in the Valencian Region, Spain.” International Journal of Environmental Research and Public Health 19, no. 16: 10366.

Ilkovich, Y. 2014. “Pulmonary Alveolar Proteinosis: A Long Way to Correct Diagnosis: Problems of Diagnostics and Therapy in Routine Practice.” Annals of Clinical and Laboratory Science 44, no. 4: 405–409.

Isono, M., M. Kokado, and K. Kato. 2022. “Why Does It Take So Long for Rare Disease Patients to Get an Accurate Diagnosis?—A Qualitative Investigation of Patient Experiences of Hereditary Angioedema.” PLoS One 17, no. 3: e0265847.

Kim, W., J. S. Cho, Y. K. Shim, et al. 2021. “Early- Onset Autosomal Dominant GTP- Cyclohydrolase I Deficiency: Diagnostic Delay and Residual Motor Signs.” Brain and Development 43, no. 7: 759–767.

Páramo-Rodríguez, L., C. Cavero- Carbonell, S. Guardiola-Vilarroig, A. López-Maside, M. E. González Sanjuán, and Ó. Zurriaga. 2023. “Demora Diagnóstica en Enfermedades Raras: Entre el Miedo y la Resiliencia.” Gaceta Sanitaria 37: 102272.

Phillips, C., A. Parkinson, T. Namsrai, et al. 2024. “Time to Diagnosis for a Rare Disease: Managing Medical Uncertainty. A Qualitative Study.” Orphanet Journal of Rare Diseases 19, no. 1: 297.

Pierucci, P., G. M. Lenato, P. Suppressa, et al. 2012. “A Long Diagnostic Delay in Patients With Hereditary Haemorrhagic Telangiectasia: A Questionnaire-Based Retrospective Study.” Orphanet Journal of Rare Diseases 7, no. 1: 33.

Qi, X., J. Xu, L. Shan, et al. 2021. “Economic Burden and Health Related Quality of Life of Ultra-Rare Gaucher Disease in China.” Orphanet Journal of Rare Diseases 16, no. 1: 358.

Rivera Gallego, A., S. Rivera García, A. Arévalo Gómez, B. Buño Ramilo, A. M. Bravo Blanco, and G. R. Suárez. 2021. “Perfil de paci- entes con enfermedades minoritarias en Galicia.” Perspectiva Desde la Medicina Interna. Galicia Clínica 82, no. Suppl 1: 9.

Rolim, A., M. C. Ribeiro, F. Diniz, et al. 2019. “Misdiagnosis and Diagnostic Delay in Non-Paraneoplastic Sensory Neuronopathies.” Arquivos de Neuro-Psiquiatria 77, no. 7: 451–455.

Tanaca, T. O. 2018. “FABRY DISEASE: THE “GREAT PRETENDER.” XXX Congresso Brasileiro de Genética Médica.” 171–172. <https://www>. sbgm.org.br/ Uploads/4Wey4KjFCi_ 04_ 02_ 2020-16_19_ 28_ 64.pdf.

Tanaka, H., and M. Shimaoka. 2023. “Trust in Physicians and Definitive Diagnosis Time Among Japanese Patients With Specific Intractable Diseases: A Cross- Sectional Study. Intractable & Rare Diseases.” Research 12, no. 2: 97–103.

Van der Kloot, W. A., N. A. Hamdy, L. C. Hafkemeijer, et al. 2010. “The Psychological Burden of an Initially Unexplained Illness: Patients With Sternocostoclavicular Hyperostosis Before and After Delayed Diagnosis.” Health and Quality of Life Outcomes 8, no. 1: 97.

Wang, K., X. Guo, S. Yu, et al. 2021. “Patient-Identified Problems and Influences Associated With Diagnostic Delay of Acromegaly: A Nationwide Cross- Sectional Study.” Frontiers in Endocrinology 12: 704496.
