# Supplementary material for: Repercussions of Diagnostic Delay in Rare Diseases
Source: J Genet Couns. 2026 Jul 17;35(4):e70258. doi: 10.1002/jgc4.70258 (PMC13379505; doi:10.1002/jgc4.70258)
Supplement: Supplementary file 4 — Table S4: Presentation of the studies limitations. [file JGC4-35-0-s003.docx]

**Supplementary Table S4 - Presentation of the studies limitations**

| STUDY | LIMITATIONS |
| --- | --- |
| 39 | Two key limitations are described due to participant bias. Firstly, since the majority of patients were aged 40 to 60, the results might not accurately reflect the experiences of younger HAE patients. Secondly, most participants reported more severe symptoms than are typical for HAE, suggesting that the findings might not be applicable to those with milder cases. |
| 65 | The authors acknowledge some limitations, for example, the calculation of diagnostic delay was limited to patients with sufficient data, potentially introducing bias. Furthermore, the recorded onset of initial symptoms could be affected by recall bias from patients, which may lead to calculation errors. |
| 69 | There weren't any limitations mentioned in the article. |
| 48 | The study has several limitations. Firstly, the small sample size made data collection challenging due to the rarity of GD. Secondly, families and patients who volunteered to participate represent a more compliant and motivated cohort, implying that the results may not be generalizable. Thirdly, the study relied on participants' memory to estimate healthcare utilization and costs, risking recall bias. Prescription drug costs were not fully included, suggesting an underestimation of GD's financial burden. Finally, the design of one question prevents the estimation of informal care costs. |
| 25 | Given the study's cross-sectional design, caution is warranted when inferring causal relationships. Participants were asked to recall experiences from the pre-diagnosis period, introducing potential recall bias. Furthermore, selection bias may be present due to the exclusion of individuals with non-confirmed diagnoses and the relatively young age of the participants. |
| 60 | Although the authors didn't explicitly address the study's limitations, they do mention that it was a retrospective study with a small sample size. |
| 6 | There weren't any limitations mentioned in the article. |
| 12 | There weren't any limitations mentioned in the article. |
| 9 | Due to the retrospective nature of the study regarding the pre-diagnostic period, recall bias may be present. This could result in two potential issues: responses may contain random elements, thus reducing their reliability, and there is a possibility that participants' current emotions influenced their recollection, leading to a blending of events from the pre-diagnostic period with those occurring before the diagnosis. |
| 34 | Given that participants were asked retrospective questions about the period preceding diagnosis, the potential for recall bias exists. Furthermore, the study focused on rare diseases as a whole, possibly resulting in a lack of representation for specific rare diseases. |
| 5 | There weren't any limitations mentioned in the article. |
| 32 | The authors identified several limitations in the study, including the lack of representation of certain rare diseases and the diverse presentations and courses of the diseases included. Changes in definitions, clinical practices, and genetic diagnostics over the study period also represent limitations. Additionally, individuals with diagnostic delays may have been more likely to participate, potentially skewing the ratio of cases to controls. |
| 33 | The article presented a few limitations, including a limited sample size, which was insufficient to enable subgroup analysis or examination of specific pathologies. Additionally, income information or financial benefits received due to incapacity were not available. |
| 74 | Due to the onset of the COVID-19 pandemic during the study's progression, the group interviews that the authors originally intended to conduct in person had to be transitioned to online platforms, potentially resulting in digital exclusion as a limitation. Additionally, as participants were required to recall past experiences, the presence of recall bias cannot be discounted. Furthermore, given that women, typically mothers, primarily served as caregivers completing the survey, gender bias may also have influenced the results. |
| 75 | The authors acknowledged the possibility of selection bias, as patient sampling was conducted in reference centers. However, upon comparing their findings with those of another study that exhibited similar results, they concluded that their sample is generalizable to other internal medicine services dealing with rare diseases. |
| 43 | There weren't any limitations mentioned in the article. |
| 64 | Since the study was conducted in a tertiary-level unit, the authors acknowledged referral bias. |
| 76 | There weren't any limitations mentioned in the article. |
| 78 | The inclusion of only hospitalized patients may have introduced selection bias. Variability in comorbidity documentation and missing clinical variables limited data quality. The retrospective design and reliance on medical records may have affected the accuracy of diagnostic delay estimates. |
| 79 | The sample included only patients with specific rare diseases, limiting generalizability. All participants were English-speaking Australians, reducing diversity. Retrospective self-reporting may have introduced recall bias, and undiagnosed individuals were excluded. |
| 80 | Non-random sampling may have introduced selection bias. Physician-reported data were subjected to recall bias. The cross-sectional design limits casual inference, and missing data reduces consistency across analyses. Findings may not generalize beyond the European context. |
| 82 | Retrospective self reporting may have introduced recall bias. Online recruitment likely caused selection bias by over representing connected individuals. The study excluded undiagnosed patients and could not track changes in diagnostic timelines over time. |
